# Respiratory mucosal immunity against SARS-CoV-2 following mRNA vaccination

**DOI:** 10.1126/sciimmunol.add4853

**Published:** 2022-07-19

**Authors:** Jinyi Tang, Cong Zeng, Thomas M. Cox, Chaofan Li, Young Min Son, In Su Cheon, Yue Wu, Supriya Behl, Justin J. Taylor, Rana Chakraborty, Aaron J. Johnson, Dante N. Schiavo, James P. Utz, Janani S. Reisenauer, David E. Midthun, John J. Mullon, Eric S. Edell, Mohamad G. Alameh, Larry Borish, William G. Teague, Mark H. Kaplan, Drew Weissman, Ryan Kern, Haitao Hu, Robert Vassallo, Shan-Lu Liu, Jie Sun

**Affiliations:** ^1^ Carter Immunology Center, University of Virginia, Charlottesville, VA, USA 22908; ^2^ Division of Infectious Disease and International Health, Department of Medicine, University of Virginia, Charlottesville, VA, USA 22908; ^3^ Division of Pulmonary and Critical Medicine, Department of Medicine, Mayo Clinic, Rochester, MN, USA 55905; ^4^ Center for Retrovirus Research, The Ohio State University, Columbus, OH, USA 43210; ^5^ Department of Veterinary Biosciences, The Ohio State University, Columbus, OH, USA 43210; ^6^ Department of Systems Biotechnology, Chung-Ang University, Anseong, Gyeonggi-do, Republic of Korea 17546; ^7^ Department of Immunology, Mayo Clinic, Rochester, MN, USA 55905; ^8^ Department of Pediatrics and Adolescent Medicine, Mayo Clinic, Rochester, MN, USA 55905; ^9^ Vaccine and Infectious Disease Division, Fred Hutchinson Cancer Research Center, Seattle, WA, USA 98109; ^10^ Department of Medicine, University of Pennsylvania, Philadelphia, PA, USA 19104; ^11^ Division of Asthma, Allergy and Immunology, Department of Medicine, University of Virginia, Charlottesville, VA, USA 22908; ^12^ Child Health Research Center, Department of Pediatrics, University of Virginia, Charlottesville, VA, USA 22908; ^13^ Department of Microbiology and Immunology, Indiana University School of Medicine, Indianapolis, IN, USA 46074; ^14^ Department of Microbiology and Immunology, University of Texas Medical Branch, Galveston, TX, USA 77555

## Abstract

SARS-CoV-2 mRNA vaccination induces robust humoral and cellular immunity in the circulation; however, it is currently unknown whether it elicits effective immune responses in the respiratory tract, particularly against variants of concern (VOCs), including Omicron. We compared the SARS-CoV-2 S-specific total and neutralizing antibody responses, and B and T cell immunity, in the bronchoalveolar lavage fluid (BAL) and blood of COVID-19 vaccinated individuals and hospitalized patients. Vaccinated individuals had significantly lower levels of neutralizing antibody against D614G, Delta (B.1.617.2) and Omicron BA.1.1 in the BAL compared to COVID-19 convalescents, despite robust S-specific antibody responses in the blood. Furthermore, mRNA vaccination induced circulating S-specific B and T cell immunity, but in contrast to COVID-19 convalescents, these responses were absent in the BAL of vaccinated individuals. Using a mouse immunization model, we demonstrated that systemic mRNA vaccination alone induced weak respiratory mucosal neutralizing antibody responses, especially against SARS-CoV-2 Omicron BA.1.1 in mice; however, a combination of systemic mRNA vaccination plus mucosal adenovirus-S immunization induced strong neutralizing antibody responses, not only against the ancestral virus but also the Omicron BA.1.1 variant. Together, our study supports the contention that the current COVID-19 vaccines are highly effective against severe disease development, likely through recruiting circulating B and T cell responses during re-infection, but offer limited protection against breakthrough infection, especially by Omicron sublineage. Hence, mucosal booster vaccination is needed to establish robust sterilizing immunity in the respiratory tract against SARS-CoV-2, including infection by Omicron sublineage and future VOCs.

## Teaser

COVID-19 mRNA vaccines induce poor anti-Omicron mucosal immunity, but this immunity can boosted with an intranasal immunization.

## INTRODUCTION

The ongoing COVID-19 pandemic is a global public health crisis, and vaccination is considered the key to ending the pandemic (*1*, *2*). It is well recognized that current SARS-CoV-2 vaccines, particularly mRNA-based vaccination, can induce robust humoral and cellular immunity and prevent severe disease caused by SARS-CoV-2 (*3*); however, protection against asymptomatic to mild infection and transmission, particularly following SARS-CoV-2 VOCs exposure, by mRNA vaccination is rather limited (*4*, *5*). The reasons for this are poorly defined.

Notably, most of the previous studies were conducted using blood to determine circulating antibodies and B and T cell immunity following vaccination (*6*). However, SARS-CoV-2 enters the host predominantly through the respiratory tract. As the result, respiratory mucosal antibodies, tissue-resident memory T (T_RM_) and B cells are likely among the early responders during viral entry, and so they are believed to be essential for the protection against the establishment of viral infection after vaccination or prior viral exposure (*7*). Thus, we reasoned that it was critical to characterize respiratory mucosal humoral and cellular immunity following COVID-19 mRNA vaccination or natural infection to better understand the vaccine- or infection-mediated protection against SARS-CoV-2 infection. Additionally, the SARS-CoV-2 Omicron sublineage, easily escapes both vaccine and infection-elicited antibody neutralization in the blood (*8*–*14*). It is currently unclear whether efficient mucosal neutralizing antibody responses can be induced by vaccination, and/or natural infection, and to what extent this could protect against SARS-CoV-2 infection.

Previous studies have examined the COVID-19 mRNA vaccine induced humoral and cellular immunity in the nasal mucosa (*15*, *16*). However, it is still controversial whether intramuscular mRNA immunization can induce meaningful neutralizing antibodies and tissue-resident T and B cells in the nasal tissue (*17*, *18*), potentially in part due to the limited amount of fluids/cells that can be sampled in nasal washes or nasal swabs. Thus, the current understanding on the COVID-19 vaccine-induced mucosal immunity in the respiratory tract remains largely elusive.

Here, we collected bronchoalveolar lavage fluid (BAL) and blood of unvaccinated healthy donors, COVID-19 vaccinated individuals and recovered hospitalized patients. We examined mucosal binding and neutralizing antibodies, and tissue-resident T and B cell responses in those subjects. Additionally, using a mouse model, we compared the mucosal immunity induced by homogeneous intramuscular mRNA vaccination versus intramuscular mRNA vaccination plus intranasal adenovirus vector booster immunization. Our results demonstrated that robust mucosal humoral and cellular immune responses were elicited in the lung by natural infection and mRNA vaccination plus adenovirus-mediated vaccination, but not by the mRNA vaccination alone.

## RESULTS

### Characterization of respiratory mucosal antibody responses following vaccination or natural infection

**Fig. 1.**
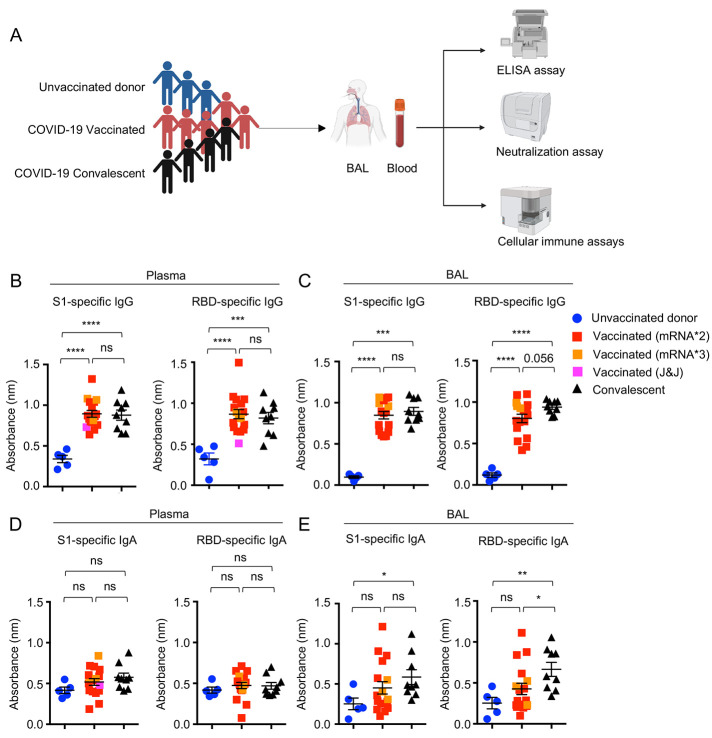
**Systemic and respiratory antibody responses in COVID-19 convalescents and vaccinated individuals. (A)** Schematic of recruited cohorts (n=5 for unvaccinated donor, n=19 for vaccinated, and n=10 for COVID-19 hospitalized convalescent) and experimental procedures. Figures were created with BioRender. **(B to E)**, Levels of SARS-CoV-2 S1 or RBD binding IgG (B and C) or IgA (D and E) in plasma and bronchoalveolar (BAL) fluid of unvaccinated donors (n=5), COVID-19 vaccinated (n=17) or convalescents (n=9). One receiving J&J was indicated as pink in the vaccinated group. Three individuals receiving the booster (BNT162b2 or mRNA-1273) were indicated as orange in the vaccinated group. Enrolled donors’ demographics were provided in Table. S1 or previous publication (*19*). Data in (B to E) are means ± SEM. Statistical differences were determined by one-way ANOVA and p values were indicated by ns, not significant (P > 0.05), * (p < 0.05), ** (p < 0.01), *** (p < 0.001), and **** (p < 0.0001).

To determine the humoral and cellular immune responses following COVID-19 vaccination, we collected blood and BAL samples from 19 COVID-19-vaccinated individuals (Fig. 1A). Most of these individuals had received two doses of mRNA vaccination, with 3 individuals receiving the third booster and one having the J&J vaccine. The vaccine type, timing of collection, age, and sex information are included in Table. S1. We compared the vaccine-induced respiratory and circulating antibodies, as well as cellular immune responses, to those of hospitalized COVID-19 convalescent patients that we have previously recruited between September 2020 to April 2021 when the D614G and Alpha variants dominated (*19*). We first performed enzyme-linked immunoassay (ELISA) to determine and compare the SARS-CoV-2 S1 or receptor binding domain (RBD)-specific IgG, IgA and IgM levels in unvaccinated control (non-SARS-CoV-2 infected), vaccinated, and convalescent groups in the plasma. Similar to what was previously shown (*3*, *20*), COVID-19 vaccination induced robust S1 or RBD-specific plasma IgG at levels comparable to severe cases of natural infection (Fig. 1B and Fig. S1A). The S1 or RBD-specific IgG levels in the BAL were also comparable between COVID-19-vaccinated and convalescent groups (Fig. 1C and Fig. S1B). Compared to unvaccinated donor, COVID-19 convalescents exhibited moderate but detectable S1-specific IgA responses in the blood (Fig. 1D and Fig. S1C). Importantly, prior severe SARS-CoV-2 infection provoked significant levels of S1 or RBD-specific IgA in the respiratory mucosa, which was not the case for COVID-19 vaccination (Fig. 1E and Fig. S1D). The lack of notable IgA production in the respiratory mucosal appeared to contrast with the detection of moderate but significant IgA responses in the saliva following mRNA vaccination (*21*, *22*). We also examined IgM in the blood and BAL, and observed that, while detectable levels of IgM were present in the circulation of both COVID-19-vaccination group and prior infection cases, only prior infection elicited significantly elevated IgM responses in the BAL (Fig. S1E-H).

Given the existence of cross-reactive and neutralizing antibodies against non-S1 or RBD epitopes (*23*–*25*), we further examined binding antibody response against Spike protein and Nucleocapsid protein, which would have informed potential unidentified infection. COVID-19 convalescents showed significantly higher S-specific IgG, IgA and N-specific IgG, but not S-specific IgM levels in blood, compared to those of vaccinated (Fig. S1 I-L). Similar results were found in the BAL (Fig. S1 M-P). Importantly, and consistent with results of S1 or RBD-specific IgA, significant level of S-specific IgA was observed both in BAL and blood from convalescents but not vaccinated individuals (Fig. S1 M, O). Together, these results revealed that, in contrast to natural infection, COVID-19 vaccination did not provoke robust IgA responses in the respiratory tract in our cohort.

### Mucosal antibody neutralizing activity against VOCs

**Fig. 2.**
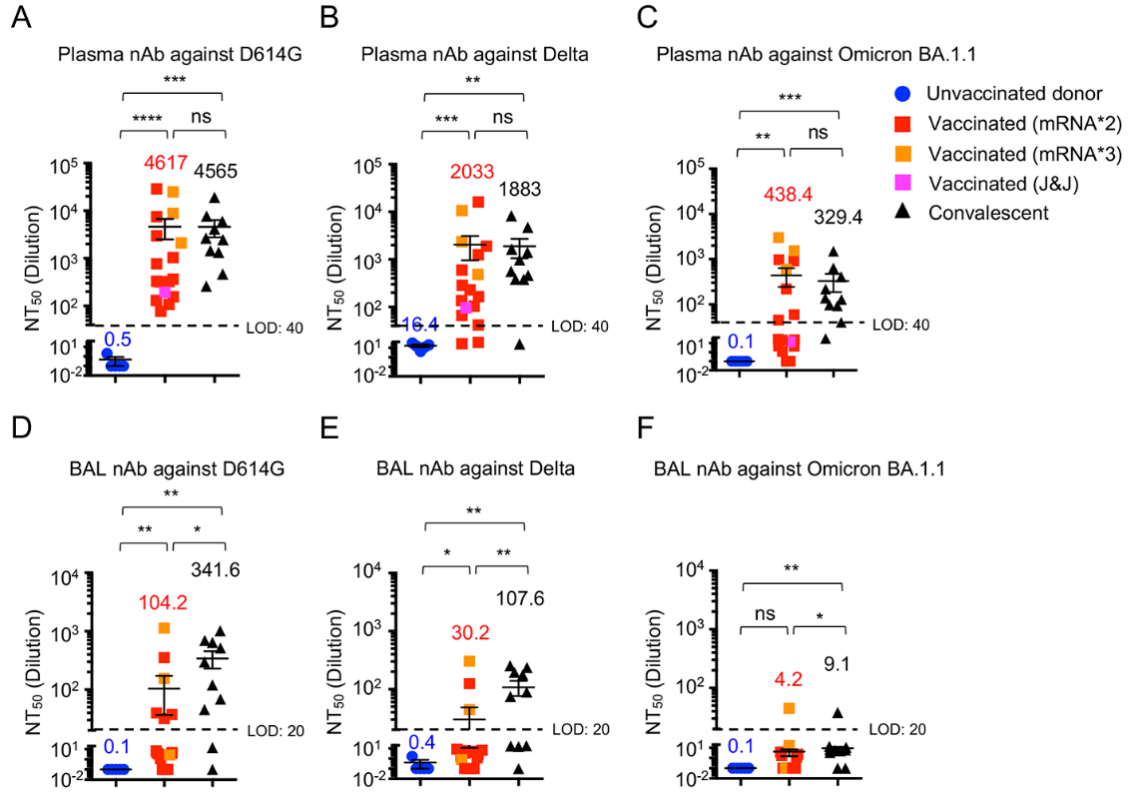
COVID-19-vaccinated individuals exhibit lower respiratory neutralizing antibody responses compared to convalescents. Plasma and BAL neutralizing activity in unvaccinated donors, vaccinated and convalescent individuals. (**A to C)** Neutralizing antibody titers (NT_50_) in plasma against SARS-CoV-2 S D614G (A) Delta (B) and Omicron BA.1.1 (C) pseudotyped virus in unvaccinated donors (n=5), vaccinated (n=17) and convalescent (n=10) individuals. HEK293T-ACE2 cells were used as targeted cells for infection. **(D to F)** Neutralizing antibody titers (NT_50_) in BAL against SARS-CoV-2 S D614G (D), Delta (E) and Omicron BA.1.1 (F) pseudotyped virus in unvaccinated donor (n=5), vaccinated (n=17) and convalescent individuals (n=10). One receiving J&J was indicated as pink in the vaccinated group. Three individuals receiving the booster shot (BNT162b2 or mRNA-1273) were indicated as orange in the vaccinated group. nAb, neutralizing antibody. LOD, limit of detection. Data are means ± SEM. Statistical differences were determined by one-way ANOVA and p values were indicated by ns, not significant (P > 0.05), * (p < 0.05), ** (p < 0.01), *** (p < 0.001), and **** (p < 0.0001).

The humoral protection against SARS-CoV-2 infection relies on the induction of robust neutralizing antibody (*26*, *27*). We thus examined the plasma neutralizing antibody activity against SARS-CoV-2 D614G, Delta and Omicron BA.1.1 spike-pseudotyped lentiviruses. While COVID-19 vaccinated and convalescent individuals exhibited comparable high levels of circulating neutralizing antibody responses against all VOCs, the Delta and Omicron BA.1.1 variants exhibited more than 2- and 10-fold decrease in neutralization titer (NT_50_), respectively, compared to D614G (Fig. 2 A-C and Fig. S2 A-E), consistent with recent results showing that VOCs, especially Omicron sublineage, have significant immune evasion capability (*8*–*14*, *28*–*30*).

We next compared neutralizing antibody responses in BALs of COVID-19-vaccinated and convalescent groups along with healthy controls. Despite the overall lower neutralizing antibody levels in BAL compared to that in the blood, the convalescent group showed ~3-fold higher neutralizing antibody activity than the vaccinated group, especially for the ancestral D614G (p < 0.05) and the Delta variant (p < 0.01) (Fig. 2D, E and Fig. S2F, G). The titers for the Omicron BA.1.1 variants were mostly below the level of detection in the BAL (Fig. 2F and Fig. S2H), reflecting the stronger escape of Omicron BA.1.1 from BAL neutralizing antibodies (Fig. S2I, J). Of note, one out of three who had received a third booster vaccination exhibited above-the-threshold yet low level of neutralization activity against Omicron BA.1.1 (Fig. 2F and Fig. S2I), suggesting that third booster vaccine may offer some, but limited, levels of protection. Overall, these results indicated that natural infection elicited stronger humoral immunity in mucosal surface compared to mRNA vaccination in our cohorts.

### Mucosal cellular immunity following vaccination or natural infection

**Fig. 3.**
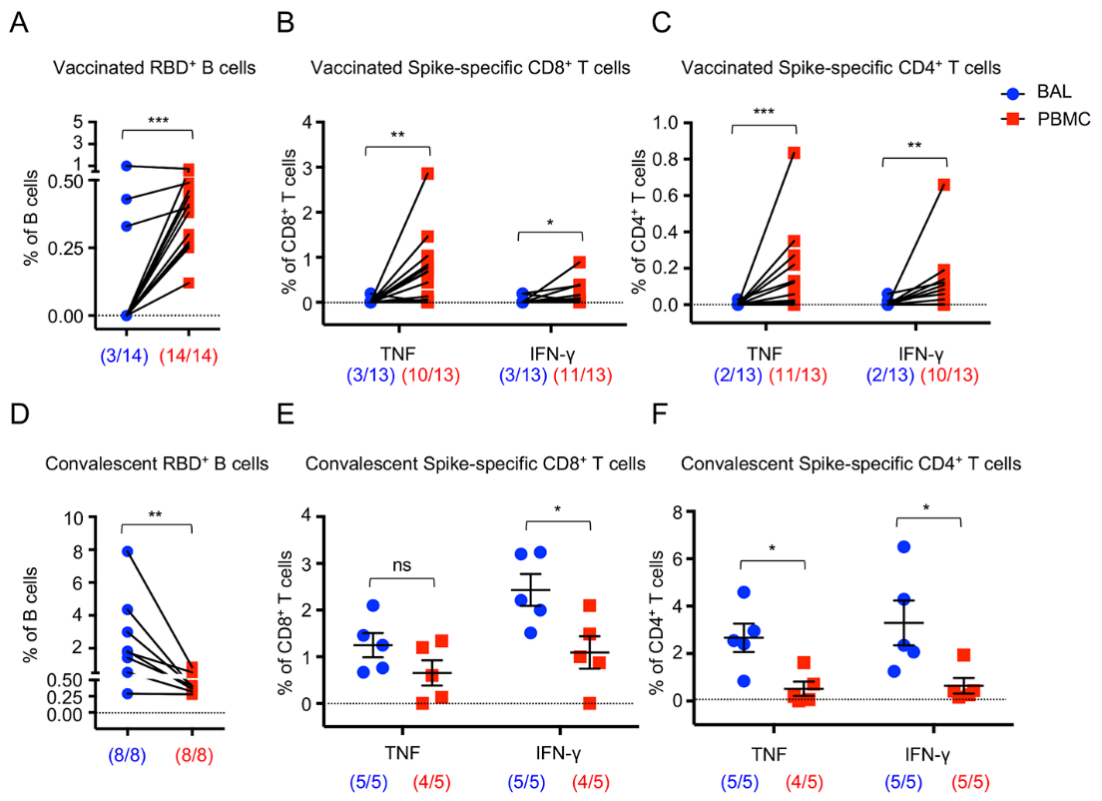
**COVID-19-vaccinated individuals exhibit systemic cellular immunity not evident in the respiratory tract. (A)** Frequency of SARS-CoV-2 RBD- specific B cells in the blood (PBMC) and the BAL of vaccinated (n=14). **(B and C)** Frequencies of TNF- and IFN-γ- producing CD8^+^ (B) or CD4^+^ (C) T cells in the blood (PBMC) and the BAL of vaccinated (n=13) after S peptide pools stimulation. **(D)** Frequency of SARS-CoV-2 RBD- specific B cells in the blood (PBMC) and the BAL of convalescent individuals (n=8). **(E and F)** Frequencies of TNF- and IFN-γ- producing CD8^+^ (E) or CD4^+^ (F) T cells in the blood (PBMC) and the BAL of convalescents (n=5) after S peptide pools stimulation. Data are means ± SEM. Numbers below the graph show ratio of positive staining within total samples. Statistical differences were determined by paired *t* test in A to D and independent *t* test in E and F. P values were indicated by ns, not significant (P > 0.05), * (p < 0.05), ** (p < 0.01), and *** (p < 0.001).

Although memory T and B cells do not confer sterilizing immunity, they are important in constraining viral dissemination and protecting against severe diseases once a virus breaches neutralizing humoral immunity (*31*–*34*). Both circulating and tissue-resident memory T and B cells are believed to provide disease protection against severe respiratory viral infection (*35*–*38*).We therefore examined systemic and tissue residing memory T and B cell responses following mRNA vaccination or natural infection. Compared to unvaccinated controls, vaccinated individuals had higher RBD-specific B cells in the blood (Fig. S3 A-D). Notably, RBD-specific B cells were markedly lower in BAL compared to those of PBMCs (Fig. 3A and Fig. S3E). As reported before (*38*, *39*), vaccination induced notable S-specific TNF or IFN-γ producing CD8^+^ or CD4^+^ T cells in the circulation but failed to elicit strong S-specific cytokine-producing CD8^+^ or CD4^+^ T cell responses in the BAL (Fig. 3B, C and Fig. S4). In contrast, convalescent BAL exhibited much higher RBD-specific B cells compared to the paired blood samples (Fig. 3D), suggesting that vaccination does not induce tissue-residing memory B cell responses as effectively as natural infection. Further, BAL from COVID-19 convalescents had higher cytokine-producing CD8^+^ and CD4 T^+^ cells than those of blood (Fig. 3E, F), although paired analysis was not performed here due to the availability of the samples obtained from a previous study (*19*). Within the total CD8^+^ or CD4^+^ T cell compartments, the levels of most memory T cell subsets in the blood and/or BAL were quite similar between unvaccinated or vaccinated individuals, except the blood central memory T cell population (Fig. S4). Thus, unlike SARS-CoV-2 natural infection, mRNA vaccination did not appear to induce significant SARS-CoV-2 specific B and T cell memory in the respiratory mucosa in contrast to that in the blood in our cohorts.

### mRNA plus mucosal Ad5-S vaccination induces strong neutralizing immunity against Omicron BA.1.1

**Fig. 4.**
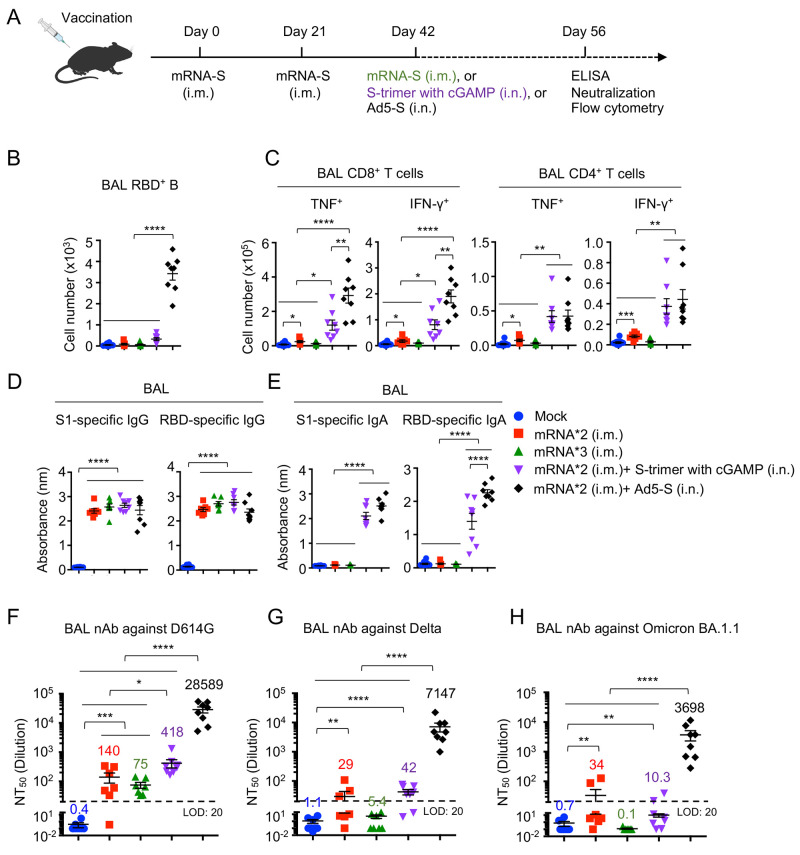
**Combination of mRNA plus mucosal adenovirus immunization induces high levels of mucosal neutralizing activity against SARS-CoV-2 Omicron BA.1.1**. C57BL/6 mice were immunized as indicated. **(A)** Schematic of vaccination strategy and experimental parameters; n=10 for PBS control (mock), n=7 for two doses of mRNA (mRNA*2 (i.m.)), n=7 for three doses of mRNA (mRNA*3 (i.m.)), n=8 for two doses of mRNA plus S-trimer with cGAMP immunization (mRNA*2 (i.m.)+ S-trimer with cGAMP (i.n.), and n=8 for two doses of mRNA plus Ad5-S immunization (mRNA*2 (i.m.)+ Ad5-S (i.n.)). **(B)** Cell numbers of RBD^+^ B in the BAL following immunization. **(C)** Cell numbers of TNF- and IFN-γ- producing CD8+ or CD4+ T in the BAL following immunization. **(D and E)** Levels of S1- and RBD- specific IgG (D) or IgA (E) were measured from BAL. **(F to H)** NT_50_ of BAL against SARS-CoV-2 S D614G (F), Delta (G) and Omicron BA.1.1 (H) pseudotyped virus were measured. i.m., intramuscular. i.n., intranasal. nAb, neutralizing antibody. LOD, limit of detection. Data are pooled from two independent experiments. Data in (B to H) are means ± SEM. Statistical differences were determined by one-way ANOVA and p values were indicated by * (p < 0.05), ** (p < 0.01), *** (p < 0.001) and **** (p < 0.0001).

Given the suboptimal mucosal immunity induced by the current COVID-19 mRNA vaccination, we used a mouse model to identify potential strategies that promote and/or amplify mucosal humoral and cellular immunity after mRNA vaccination. To this end, we immunized wild type C57BL/6 mice with PBS, two doses of mRNA-encoding codon-optimized S (mRNA-S), three doses of mRNA-S, two doses of mRNA-S plus an intranasal immunization of S protein trimer with adjuvant (STING ligand, cGAMP (*40*)), or two doses of mRNA-S plus an intranasal of adenovirus type 5 encoding S protein (Ad5-S) (Fig. 4A). We focused on intranasal immunization in mRNA-immunized mice, in keeping the contention that induction of mucosal immunity likely occurs in previously vaccinated individuals who will be willing to receive mucosal booster vaccines. mRNA plus Ad5-S vaccination induced greatly increased BAL RBD-specific B cells (Fig. 4B). Furthermore, mRNA plus Ad5-S vaccination induced potent mucosal CD8^+^ and CD4^+^ T cell responses but not in the spleen, while mRNA plus cGAMP/S trimer immunization led to robust CD4^+^ T cell responses in the spleen (Fig. 4C and Fig. S5). mRNA immunization, with or without the third dose of mucosal immunization, induced strong circulating S1 or RBD-specific IgG in the blood and the BAL (Fig. 4D and Fig. S6A). A third dose of mucosal immunization of S protein, with S trimer plus cGAMP or Ad5-S, resulted in significant increases of both S1 and RBD-specific IgA in the BAL (Fig. 4E), with Ad5-S inducing the highest RBD-specific IgA in the respiratory mucosa (Fig. 4E). Ad5-S also generated significantly higher levels of plasma IgA, IgM and BAL IgM than other groups (Fig. S6 B-D).

All immunized groups showed strong neutralization against D614G and the Delta variant in the plasma, although three-dose mRNA, or two-dose mRNA plus Ad5-S, vaccination induced higher levels of neutralizing antibody compared to two doses of mRNA immunization (Fig. S6E, F). As would be expected, the mouse plasma neutralization activities against Omicron BA.1.1 were also dramatically reduced relative to D614G or Delta (Fig. S6G), indicating that Omicron BA.1.1 is capable of escaping immunization-induced neutralizing antibody responses in the mouse blood similar to that in humans. However, we were still able to detect neutralizing antibody activities, at approximately similar levels, against the Omicron BA.1.1 variant in all immunized groups (Fig. S6G).

The neutralizing antibody activity in the BAL of mRNA immunized mice (two doses or three doses) were generally lower than those in the blood, but clearly detectable against D614G, with ~4-fold reduction in Delta, yet were around the limit of detection for the Omicron BA.1.1 variant (Fig. 4 F-H). Strikingly, mRNA plus Ad5-S significantly increased the neutralization titer against the ancestral D614G by approximately 3 logs compared to other vaccination groups, and more importantly, maintained the strong neutralization activity against Delta as well as the Omicron BA.1.1 variant (Fig. 4 F-H).

To confirm that the IgA response is induced by boosting the primed response, we immunized wild type C57BL/6 mice with PBS, Ad5-S alone, or one dose mRNA-S plus Ad5-S. The S1 or RBD-specific IgA levels were generally higher in plasma from mRNA-S plus Ad5-S immunization, compared to those of Ad5-S immunization alone (Fig. S7A). Of note, mRNA plus Ad5-S immunization induced dramatically higher S1 or RBD-specific IgA level in the BAL, but not in nasal washes, compared to those of Ad5-S immunization alone (Fig. S7B, C). Furthermore, S1 or RBD- specific IgG and IgM levels were higher in plasma, BAL, as well as nasal wash after mRNA-S plus Ad5-S immunization compared to those of Ad5-S immunization alone (Fig. S7 D-I). Additionally, mRNA-S plus Ad5-S immunization induced strong antigen-specific T cell responses, particularly in the BAL (Fig. S7 J-N). Intranasal vaccination of anesthetized mice inadvertently introduces vaccine material into lower lungs (*41*); however, Ad5-S i.n. immunization did not appear to induce notable weight loss and provoked relatively moderate inflammatory responses in the lung compared to those of influenza infection (Fig. S8), suggesting that mucosal adenovirus delivery does not seem to lead to significant host morbidity or overt lung pathology.

Altogether, these data indicated that compared to systemic mRNA booster, the mucosal Ad5-S booster immunization elicits broadened antibody neutralization in the BAL against VOCs. Thus, we have here identified a promising immunization strategy that can induce potent mucosal neutralizing antibody effectively against the Omicron BA.1.1 variant (Fig. S9).

## DISCUSSION

COVID-19 mRNA vaccination elicited at least comparable neutralizing antibody levels as COVID-19 convalescents in the circulation but generated considerably lower mucosal IgA and neutralizing antibody responses against SARS-CoV-2 D614G, Delta and Omicron BA.1.1 variants than those of convalescents, indicating that the overall magnitude of mucosal antibody responses is suboptimal following vaccination. Consistent with the idea, several recent large clinical studies have revealed that mRNA vaccination is relatively less efficient in the protection against infection compared to prior natural infection during the Delta wave (*42*–*44*). Of note, the Omicron BA.1.1 variant almost completely escaped the neutralization activity of BAL from either vaccinated or previously infected individuals. Additionally, we provide compelling real-world evidence that mRNA vaccination does not induce notable lung tissue-residing S-specific memory B and T cells. Thus, despite the induction of robust circulating humoral and cellular immunity, current COVID-19 mRNA vaccines likely do not provoke sufficient levels of mucosal immunity in the human lower respiratory tract that would be needed for immediate clearance of the infectious Omicron BA.1.1 variant to prevent the establishment of infection. Such a notion is consistent with the fact that the Omicron sublineage continues to spread at a rapid pace in regions with high rates of vaccination and/or prior natural infection.

Our data do not dispute the notion that current vaccines are highly effective in preventing hospitalization and death. The prevention of severe disease after infection is conferred mainly by memory T and B cells (*38*, *45*). To this end, CD8 T cell epitopes within Omicron Spike protein remain conserved to those of ancestral strains (*46*–*48*). Thus, even though Omicron is able to breach the defense of mucosal neutralizing antibody to cause infection, the recruitment of vaccine-induced circulating memory T cells during SARS-CoV-2 breakthrough infection enables protection that restrains further viral dissemination, preventing severe disease development following infection. Nevertheless, these data suggest that mucosal humoral immunity is particularly vulnerable for immune escape by Omicron BA.1.1 and other sublineage. It is thus quite likely that the current vaccine strategy, even with further boosters, will not achieve “herd immunity” or prevent the occurrence of new infections or re-infections with future VOCs, particularly those with immune-evasive properties like Omicron sublineage. Thus, our findings have significant public health implications.

Our data suggest that a mucosal SARS-CoV-2 booster vaccine may be necessary to achieve more robust immunity and protection from re-infection by future variants. To this end, we have provided a proof-of-principle experiment that systemic mRNA plus mucosal Ad5-S vaccination provoked strong cellular immunity in the respiratory tract, and compelling mucosal IgA and neutralizing activity against Omicron BA.1.1. Mucosal adenovirus delivery has concerns of safety and applicability on a large scale. However, an Ad5-S based mucosal booster strategy in vaccinated individuals has been found safe and induced stronger plasma antibody responses (*49*). Thus, an adenoviral booster vaccine potentially has great translational and clinical relevance. Alternatively, Emerging novel vaccine platforms like virus-like nanoparticles (*50*), which can provide strong adjuvant activity and prolonged antigen presentation in vivo, may also be a promising approach to boost mucosal neutralizing immunity against Omicron or future VOCs.

Compared to convalescents, BAL from vaccinated individuals had reduced neutralizing activities despite similar levels of S1 or RBD-specific IgG present in the two groups. Further, BAL from mice immunized with mRNA alone or mRNA plus S-trimer had comparable RBD-specific IgG levels to those of mRNA plus Ad5-S immunized mice, with the latter showing markedly higher neutralizing activities against SARS-CoV-2 ancestral virus or VOCs, indicating that BAL IgG levels alone do not perfectly correlate with the levels of neutralizing activity. Whether this is due to the strong RBD-specific IgA responses present in the respiratory mucosa following natural infection or mucosal Ad5-S booster immunization is currently unknown. Of note, prior studies have identified that viral infection can lead to persistent germinal center reaction and antibody production in the lung (*51*, *52*). Therefore, local antigen-specific IgG or IgA produced in situ in the respiratory tract following viral infection may provide better neutralizing activities than those diffused solely from the blood following systemic immunization. In addition, persistent damage, inflammation, or chronic antigen deposition in the lung may further facilitate the development of local neutralizing antibody responses following natural infection.

Our study has several limitations. Due to the highly invasive nature of the BAL procedure, we were not able to recruit a large cohort of study participants. Furthermore, the study procedure made it challenging to time recruitment or perform a longitudinal analysis; rather it enabled a snapshot of vaccination or infection-induced mucosal immunity. Additionally, most of the participants were older and may not be representative of the entire vaccinated population, although this age group is considered as the primary targeting population for vaccination as they are at highest risk of infection associated with mortality and complications. Finally, soluble Spike trimers engaging ACE2 may trigger undesirable side effects following immunization of adenovirus-vectored vaccine (*53*); thus, using Spike harboring mutations known to abolish high-affinity interactions with human ACE2 shall be considered for future vaccine design.

Nevertheless, we have provided critical evidence detailing the mucosal humoral and cellular immunity following vaccination in the respiratory tract. Our study highlights the importance of focusing on vaccine-induced mucosal immunity (*54*) and argues for the necessity of a mucosal booster strategy in addition to the current approach of intramuscular COVID-19 vaccines.

## METHODS AND MATERIALS

### Study design

The goal of the study was to identify the respiratory mucosal immune response following COVID-19 vaccination. We recruited a cohort of unvaccinated healthy individuals (n=5) and COVID-19 vaccinated individuals (n=19), most of which receiving mRNA vaccination, as well as convalescents (n=10) who were recovered from acute COVID-19 for 2 to 3 months. We obtained blood samples and BAL fluid from the study subjects. ELISA and viral neutralization assay were performed to determine SARS-CoV-2–specific binding and neutralizing antibodies in the circulation or in the respiratory tract. Spectral flow cytometry was performed with PBMCs and BAL cells for the characterization of circulating and respiratory adaptive immune cell responses in this cohort. Lastly, we used an animal immunization model for the development of an intranasal booster strategy that can induce robust mucosal immune response in the respiratory tract, particularly against SARS-CoV-2 VOCs.

### Study cohorts

BAL or blood samples were collected from unvaccinated donors, COVID-19 vaccinated individuals, or COVID-19 convalescents at Mayo Clinic under protocols approved by Mayo Clinic Institutional Review Boards (protocol ID 19-012187). Study participants included non-pregnant adults who were undergoing flexible bronchoscopy as part of their clinical management. However, participants who had presence of hereditary respiratory diseases (such as cystic fibrosis), clinical history of primary aspiration, neuromuscular problems, primary or secondary immune deficiencies, invasive viral or bacterial infections or a cancer diagnosis were excluded in the study. Informed consent for the use of BAL, blood and their derivatives for research was obtained from all enrolled individuals. For COVID-19 convalescents, three unvaccinated and three vaccinated samples were from a cohort that were previously recruited (*19*). Most of the vaccinated subjects received two doses of Pfizer/bioNTech (BNT162b2) or Moderna (mRNA-1273) mRNA vaccination, with three individuals receiving the third booster vaccination and one individual having the J&J (Ad26.COV2.S) vaccination. All vaccinated samples were obtained within 8 months post vaccination. Full cohort and demographic information are provided in **Table. S1**.

### BAL collection

Fiberoptic bronchoscopy and BAL were performed using moderate conscious sedation using standard clinical procedural guidelines in an outpatient bronchoscopy suite. Conscious sedation was administered in accordance with hospital policies, and a suitably trained registered nurse provided monitoring throughout the procedure. The bronchoscope was wedged (tip of the scope placed securely) in an airway leading a segment of the lung. About 100 to 200 mL of saline were instilled in 20-mL aliquots until 60 mL of lavage fluid was obtained. The specimen was placed on ice and immediately hand carried to laboratory for analysis. The fluid collected was placed on ice and transferred immediately to the laboratory for processing.

### Human single-cell purification

Plasma was isolated from whole blood by centrifuging at 1,600 rpm, room temperature (RT), for 10 min. Plasma was collected and inactivated for 30 min at 56°C, then stored at −80°C for ELISA and neutralization assay. After plasma isolation, leftover blood was mixed with phosphate-buffered saline (PBS) and then gently put over on Ficoll-Paque (Cytiva, 17144002) in a 15 mL tube. Buffy coat generated by centrifuging at 400 g for 30 min at RT was collected. For single-cell purification from BAL, BAL was filtered with a 70-μm cell strainer (Falcon) and then centrifuged at 350 g for 6 min at 4°C. Supernatant was collected and aliquots were stored at −80°C for ELISA and neutralization assay. Supernatant of BAL was further concentrated for 20x using 3 kDa Amicon Ultra-15 Centrifugal Filter Unit (Millpore Sigma, UFC900324) before use. The cells were collected for flow cytometry analysis.

### Mice immunization and sample collection

Antigens encoded by the mRNA vaccines were derived from SARS-CoV-2 isolate Wuhan-Hu-1 (GenBank MN908947.3). Nucleoside-modified mRNA encoding the full length of the Spike protein from SARS-CoV-2 with two proline mutations (mRNA-S) were synthesized by in vitro transcription using T7 RNA polymerase (MegaScript, Ambion) as previously reported (*55*). mRNAs were formulated into lipid nanoparticles (LNP) using an ethanolic lipid mixture of ionizable cationic lipid and an aqueous buffer system as previously reported (*56*). Formulated mRNA-LNPs were prepared according to RNA concentrations (~1μg/μL) and were stored at -80°C for animal immunizations. All animal protocols were approved by the Institutional Animal Care and Use Committees (IACUC) of the Mayo Clinic (Rochester, MN, #A00002035) or the University of Virginia (Charlottesville, VA, #4369). 8- to 10-week-old female C57BL/6 mice (The Jackson Lab, 000664) were vaccinated with one dose or two doses of 1 μg mRNA-S with a 21-day interval. Another 21 days later, mice were boosted with PBS, 1 μg mRNA-S intramuscularly, 3 μg S-trimer (Sino Biological, 40589-V08H8) adjuvanted with 10 μg 2'3′-cGAMP (Invivogen, tlrl-nacga23) intranasally, or 10^9^ pfu adenovirus type 5 encoding S protein (Ad5-S) (University of Iowa Viral Vector Core) intranasally after anesthetized by intraperitoneal injection of ketamine and xylazine. The volume of intranasal administrations was 30 μL. Three doses of PBS administered mice were used as control. 14 days later, mice were euthanatized. BAL, blood and splenocytes were collected for analysis. Isolated plasma inactivated for 30 min at 56°C, supernatant of the first 600 μL (for two doses mRNA plus i.n. 3^rd^ immunization) or 1.6 mL (for one dose mRNA plus 2^nd^ i.n. immunization), and Nasal wash of 1 mL collected were stored at −80°C for ELISA or neutralization assay. The cells were collected for flow cytometry analysis. 200 pfu Influenza A/PR8/34 were used to infect mice intranasally. At day 6 post infection, the left lobe of the lung was subjected for histopathology.

### Binding antibody response against SARS-CoV-2

General ELISA method has been previously described (*19*). Briefly, recombinant SARS-CoV-2 proteins including RBD (Sino Biological, 40592-V08H), spike S1 D614G (S1) (Sino Biological, 40591-V08H3), spike S1+S2 ECD (S) (Sino Biological, 40589-V08H4), or nucleocapsid protein (N) (GenScript, Z03488) were precoated to 96-well plates overnight at 4°C. The following day, plates were washed with wash buffer (0.05% Tween 20 in PBS) and then blocked with Assay dilution buffer (Biolegend, 421203) for 1 hour at RT. Plasma or 20x concentrated BAL from unvaccinated donors, vaccinated and convalescents were diluted in “Assay dilution buffer” starting at a 1:5 or 1:1 dilution, respectively, and then serially diluted by a factor of 5. Plasma from mice were diluted starting at 1:100 dilution, and then serially diluted by a factor of 5. BAL from mice was not concentrated or diluted. Samples were added to the plate and incubated for 2 hours at RT. After washing three times with wash buffer, secondary antibodies diluted with “Assay dilution buffer” were added to the plate and then incubated for 1 hour at RT. Secondary antibodies including anti-human IgG (Sigma-Aldrich, A6029), anti-human IgA (Hybridoma Reagent Laboratory, HP6123), anti-human IgM (Sigma Aldrich, A6907), anti-mouse IgG (SouthernBiotech,1030-05), anti-mouse IgA (SouthernBiotech, 1040-05), anti-mouse IgM (SouthernBiotech, 1020-05) were diluted as indicated respectively. Plates were washed three times and then developed with 3,3′,5,5′ tetramethyl benzidine (TMB) buffer (Thermo Fisher Scientific, 00-4201-56) for 10 min at RT. Sulfuric acid (2 M) was used as STOP buffer. Plates were read at about 5 min on a microplate reader (Molecular Devices) at 450 nm with SoftMax Pro Software. The optical density (OD) value at 1:5 dilution for human plasma, 1:1 dilution for human BAL, 1:100 for mice plasma (1:500 for IgG), or original mice BAL and Nasal wash were displayed, respectively; one dot represents each individual.

### Neutralizing antibody response against SARS-CoV-2

Pseudovirus neutralization assays were performed as previously reported (*57*). Briefly, in a 96-well format, plasma or BAL were diluted starting at a 1:40 or 1:20 dilution, respectively, and then serially diluted by a factor of 4. The pseudoviruses including D614G, Delta (B.1.617.2) and Omicron BA.1.1 were incubated with plasma or BAL for 1 hour at 37°C, followed by infection of 2x10^4^ pre-seeded HEK293T-ACE2 cells (BEI, NR-52511) on a 96-well polystyrene tissue culture plate. Gaussia luciferase activity in cell culture media was assayed 48 hours and 72 hours after infection. Note that, to ensure valid comparisons between SARS-CoV-2 variants, equivalent amounts of pseudovirus were used based on the pre-determined virus titers and samples of different variants were loaded side by side in each plate. Neutralizing titer 50% (NT_50_) for each sample was determined by non-linear regression with least squares fit in GraphPad Prism 5 (GraphPad Software).

### Flow Cytometry analysis

Fresh mice and human cells or frozen human PBMC or BAL cells recovered and rested overnight in 37°C, 5% CO_2_ incubator, were washed with FACS buffer (1% FBS and 0.5 M EDTA in PBS), then stained with antibodies as listed in **Table. S2** for human and **Table. S3** for mice. Intracellular Cytokine Staining (ICS) was performed to detect vaccine-specific T cell response. Briefly, Cells were washed with FACS buffer and resuspended with complete RPMI with 10 mM HEPES supplemented with 10% FBS, 2-Mercaptoethanol, Sodium Pyruvate, Non-Essential Amino Acids, Pen-Strep, and L-Glutamine. Cells were then stimulated with 1 μg/mL S peptide pool (JPT, PM-WCPV-S) for stimulation for 6 hours (PBMC for 16 hours). In the last 4 hours of incubation, protein transport inhibitor Brefeldin-A was added. Cells stimulated with PMA/Ionomycin or DMSO only were included as positive control and negative control, respectively. Following stimulation, cells were first stained for surface markers on ice for 30 min. After washing with PBS, cells were resuspended with Zombie-dye for viability staining and incubated at room temperature (RT) for 15 min. Following surface and viability staining, cells were fixed with fixation buffer (Biolegend, 420801) and permeabilized with perm/wash buffer (Biolegend, 421002), followed by intracellular cytokine staining on ice for 30 min. Cells were then washed with perm/wash buffer and resuspended with FACS buffer. To detect RBD-specific B cells, recombinant RBD proteins coupled with PE and APC were incubated with the cells for 30 min at 4°C. RBD-PE and RBD-APC double-positive B cells were identified as RBD^+^ B cells. To detect S_539-546_ epitope specific CD8^+^ T cells, SARS-CoV-2 S_539-546_ MHC class I tetramer (H-2K_b_) (NIH Tetramer Core) was incubated with the cells for 30 min at 4°C. CD44^+^ Tetramer^+^ positive CD8^+^ T cells were identified as S_539-546_ epitope specific CD8^+^ T cells. Cell population data were acquired on a multi-spectral flow cytometer Cytek Aurora (Cytek Biosciences) or Attune NxT (Thermo Fisher Science) and analyzed using FlowJo Software (10.8.1, Tree Star). All human data from cytokines production assay were background-subtracted using paired DMSO treated control samples.

### Histopathology

At 6 days post infection with Ad5-S or PR8, left lobe of the whole lung from mice was harvested and fixed in formalin (Thermo Fisher Scientific) until embedding. Fixed lung tissues were embedded in paraffin. lung tissue slides were stained with hematoxylin and eosin by University of Virginia Research Histology Core (Charlottesville, VA), and scanned by University of Virginia Biorepository and Tissue Research Facility (BTRF) (Charlottesville, VA).

### Statistical analysis

Statistical tests are indicated in the corresponding figure legends. One way ANOVA was used in multi group comparison. Paired *t* test was used in PBMC and BAL paired comparison. Others were analyzed using independent *t* test. All tests were performed with a nominal significance threshold of P < 0.05, which displayed by a single asterisk. P > 0.05 was displayed by ns, means not significant. Two asterisks indicate P < 0.01, Three asterisks indicate P < 0.001, four asterisks indicate P < 0.0001.
