# Supplementary material for: Respiratory mucosal immunity against SARS-CoV-2 following mRNA vaccination
Source: Sci Immunol. 2022 Jul 19:eadd4853. doi: 10.1126/sciimmunol.add4853 (PMC9348751; doi:10.1126/sciimmunol.add4853)
Supplement: Supplementary file 1 — Figs. S1 to S9 Tables S1 to S3 [file sciimmunol.add4853_sm.pdf]

Science Immunology
AAAS

Supplementary Materials for

Respiratory mucosal immunity against SARS-CoV-2 following mRNA vaccination

Jinyi Tang *et al.*

Corresponding authors: Shan-Lu Liu, liu.6244@osu.edu; Jie Sun, js6re@virginia.edu

DOI: 10.1126/sciimmunol.add4853

The PDF file includes:

Figs. S1 to S9
Tables S1 to S3

Other Supplementary Material for this manuscript includes the following:

Table S4

Respiratory mucosal immunity against SARS-CoV-2 following mRNA vaccination

Jinyi Tang[1,2,3*], Cong Zeng[4,5*], Thomas M. Cox[3], Chaofan Li[1,2,3], Young Min Son[1,6], In Su Cheon[1,2,3], Yue Wu[7], Supriya Behl[8], Justin J. Taylor[9], Rana Chakaraborty[8], Aaron J. Johnson[7], Dante N Shiavo[3], James P. Utz[3], Janani S. Reisenauer[3], David E. Midthun[3], John J. Mullon[3], Eric S. Edell[3], Mohamad G. Alameh[10], Larry Borish[11], William G. Teague[12], Mark H. Kaplan[13], Drew Weissman[10], Ryan Kern[3], Haitao Hu[14], Robert Vassallo[3], Shan-Lu Liu[4,5#] and Jie Sun[1,2,3,7#]

1. Carter Immunology Center, University of Virginia, Charlottesville, VA, USA 22908
2. Division of Infectious Disease and International Health, Department of Medicine, University of Virginia, Charlottesville, VA, USA 22908
3. Division of Pulmonary and Critical Medicine, Department of Medicine, Mayo Clinic, Rochester, MN, USA 55905
4. Center for Retrovirus Research, The Ohio State University, Columbus, OH, USA 43210
5. Department of Veterinary Biosciences, The Ohio State University, Columbus, OH, USA 43210
6. Department of Systems Biotechnology, Chung-Ang University, Anseong, Gyeonggi-do, Republic of Korea 17546
7. Department of Immunology, Mayo Clinic, Rochester, MN, USA 55905
8. Department of Pediatrics and Adolescent Medicine, Mayo Clinic, Rochester, MN, USA 55905
9. Vaccine and Infectious Disease Division, Fred Hutchinson Cancer Research Center, Seattle, WA, USA 98109
10. Department of Medicine, University of Pennsylvania, Philadelphia, PA, USA 19104
11. Division of Asthma, Allergy and Immunology, Department of Medicine, University of Virginia, Charlottesville, VA, USA 22908
12. Child Health Research Center, Department of Pediatrics, University of Virginia, Charlottesville, VA, USA 22908
13. Department of Microbiology and Immunology, Indiana University School of Medicine, Indianapolis, IN, USA 46074
14. Department of Microbiology and Immunology, University of Texas Medical Branch, Galveston, TX, USA 77555

*, # These authors contribute equally

Email: liu.6244@osu.edu, js6re@virginia.edu

Supplementary Materials

Fig. S1. SARS-CoV-2 binding IgG, IgA and IgM responses in human plasma and BAL.

Fig. S2. SARS-CoV-2 neutralizing antibody responses in human plasma and BAL against D614G, Delta, and Omicron BA.1.1.

Fig. S3. B cell response in human PBMC and BAL.

Fig. S4. T cell response in human PBMC and BAL.

Fig. S5. T cell responses in mouse BAL and spleen.

Fig. S6. Antibody responses in mouse plasma and BAL.

Fig. S7. Antibody and T cell responses in mouse following mRNA-S plus Ad5-S or Ad5-S alone vaccination.

Fig. S8. Lung pathology following intranasal Ad5-S or influenza infection.

Fig. S9. Model on the mechanism of protection by intramuscular mRNA vaccine-induced adaptive immunity against severe diseases, rather than infection.

Table. S1. Enrolled donors.

Table. S2. List of human antibodies used for flow cytometry.

Table. S3. List of mouse antibodies used for flow cytometry.

Table. S4. Raw data.

Supplementary Materials

Figure S1

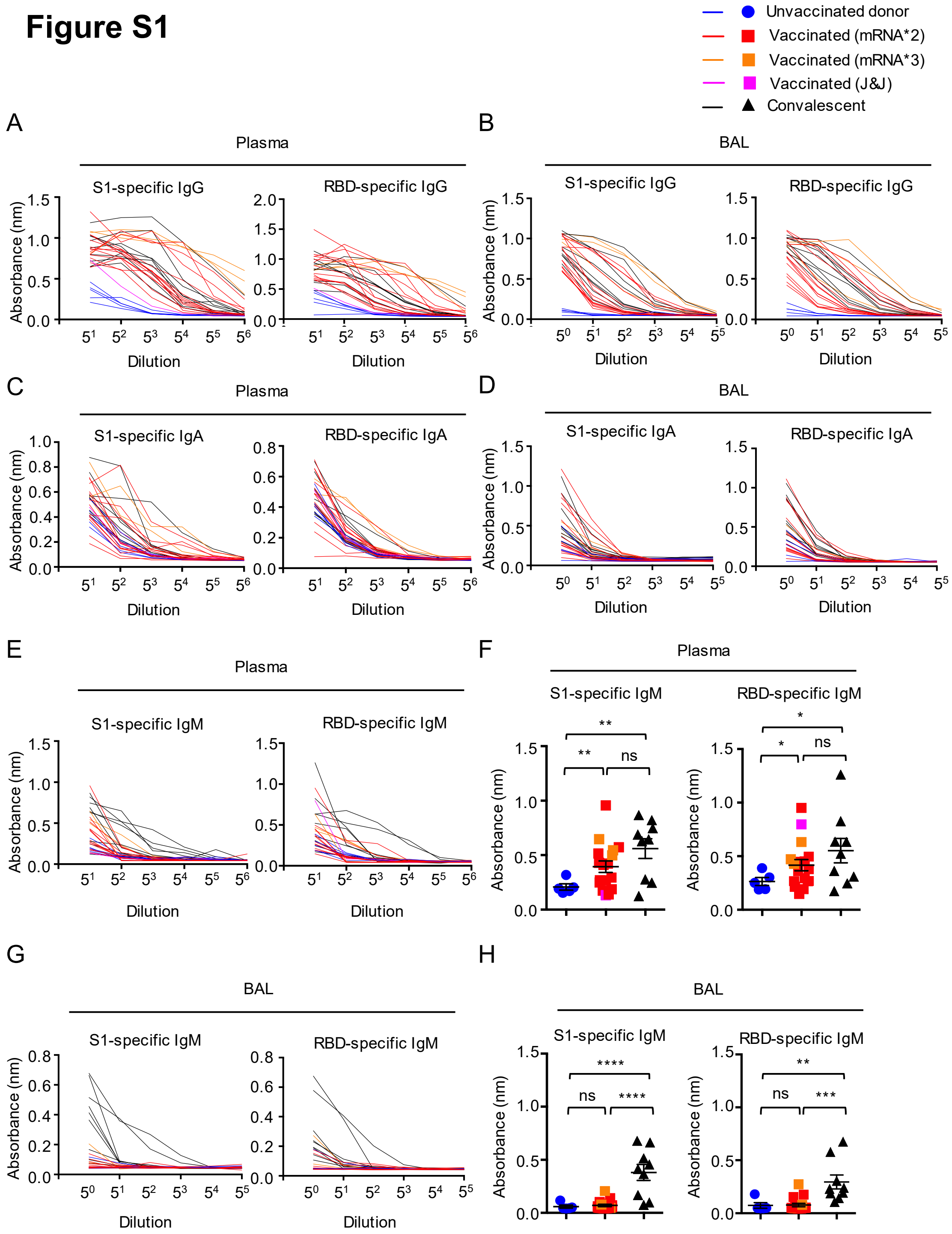

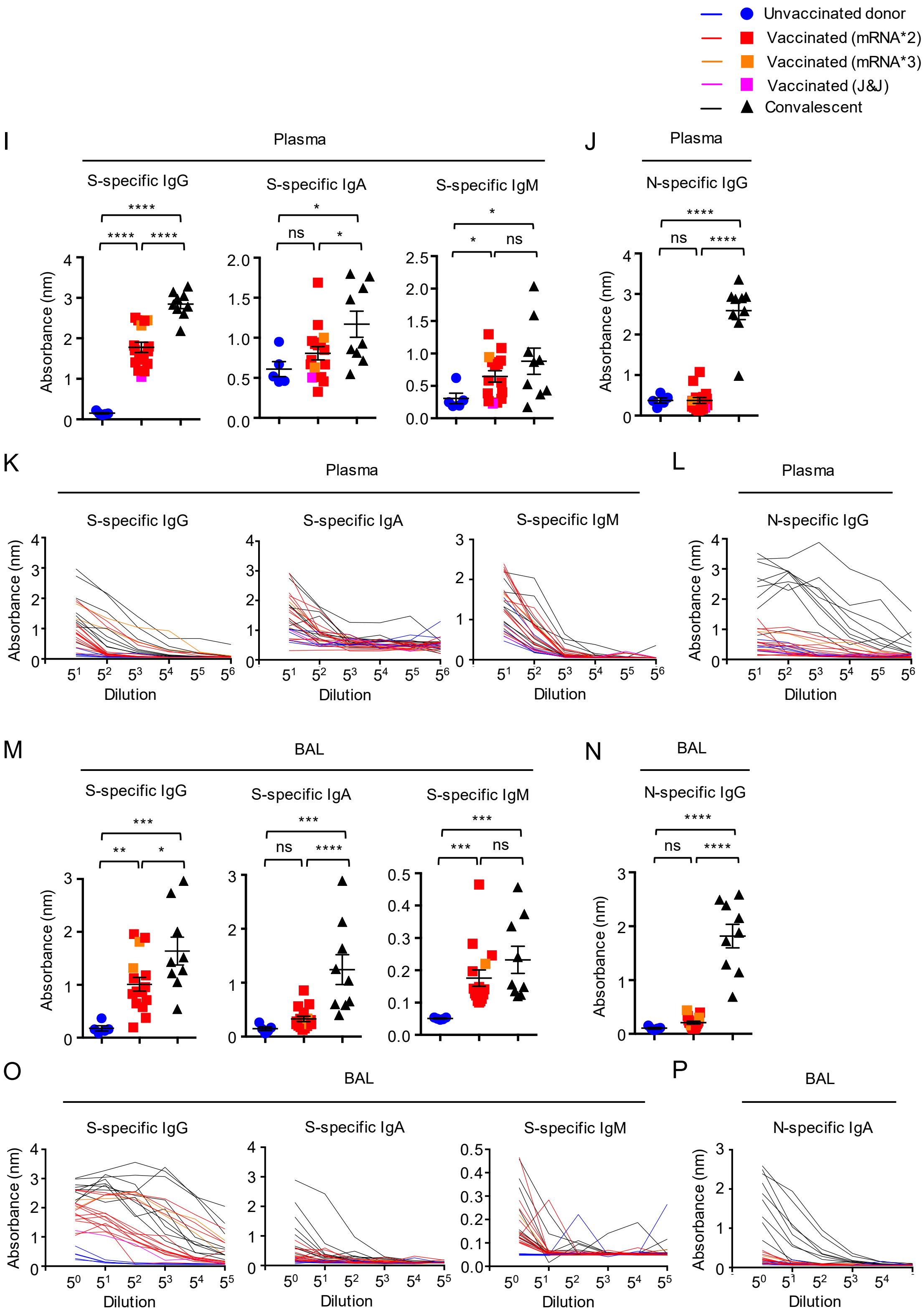

Unvaccinated donor
Vaccinated (mRNA*2)
Vaccinated (mRNA*3)
Vaccinated (J&J)
Convalescent
I
Plasma
S-specific IgG
S-specific IgA
S-specific IgM
J
Plasma
N-specific IgG
Absorbance (nm)
K
Plasma
S-specific IgG
S-specific IgA
S-specific IgM
L
Plasma
N-specific IgG
Dilution
M
BAL
S-specific IgG
S-specific IgA
S-specific IgM
N
BAL
N-specific IgG
O
BAL
S-specific IgG
S-specific IgA
S-specific IgM
P
BAL
N-specific IgA
Absorbance (nm)
Dilution

Fig. S1. SARS-CoV-2 binding IgG, IgA and IgM responses in human plasma and BAL. (A and B) Levels of SARS-CoV-2 S1 or RBD binding IgG in the plasma (A) and bronchoalveolar (BAL) fluid (B) from unvaccinated donors, COVID-19 vaccinated or convalescents. **(C and D)** Levels of SARS-CoV-2 S1 or RBD binding IgA in the plasma (C) and BAL (D) from unvaccinated donors, COVID-19 vaccinated or convalescents. (**E and F)** Levels of SARS-CoV-2 S1 or RBD binding IgM in the plasma from unvaccinated donors, COVID-19 vaccinated or convalescents. **(G and H)** Levels of SARS-CoV-2 S1 or RBD binding IgM in the BAL from unvaccinated donors, COVID-19 vaccinated or convalescents. **(I to P)** Levels of SARS-CoV-2 S binding IgG, IgA and IgM, or N binding IgG in the plasma (I to L) and BAL (M to P) from unvaccinated donors, COVID-19 vaccinated or convalescents. n=5 for unvaccinated donors, n=17 for COVID-19 vaccinated, n=10 for COVID-19 convalescents. Enrolled donors' demographics provided in Table. S1. Data in F, H, I, J, M and N are means ± SEM. Statistical differences were determined by one-way ANOVA and p values were indicated by ns, not significant ($P > 0.05$), * ($p < 0.05$), ** ($p < 0.01$), ***($p < 0.001$) and **** ($p < 0.0001$).

Figure S2

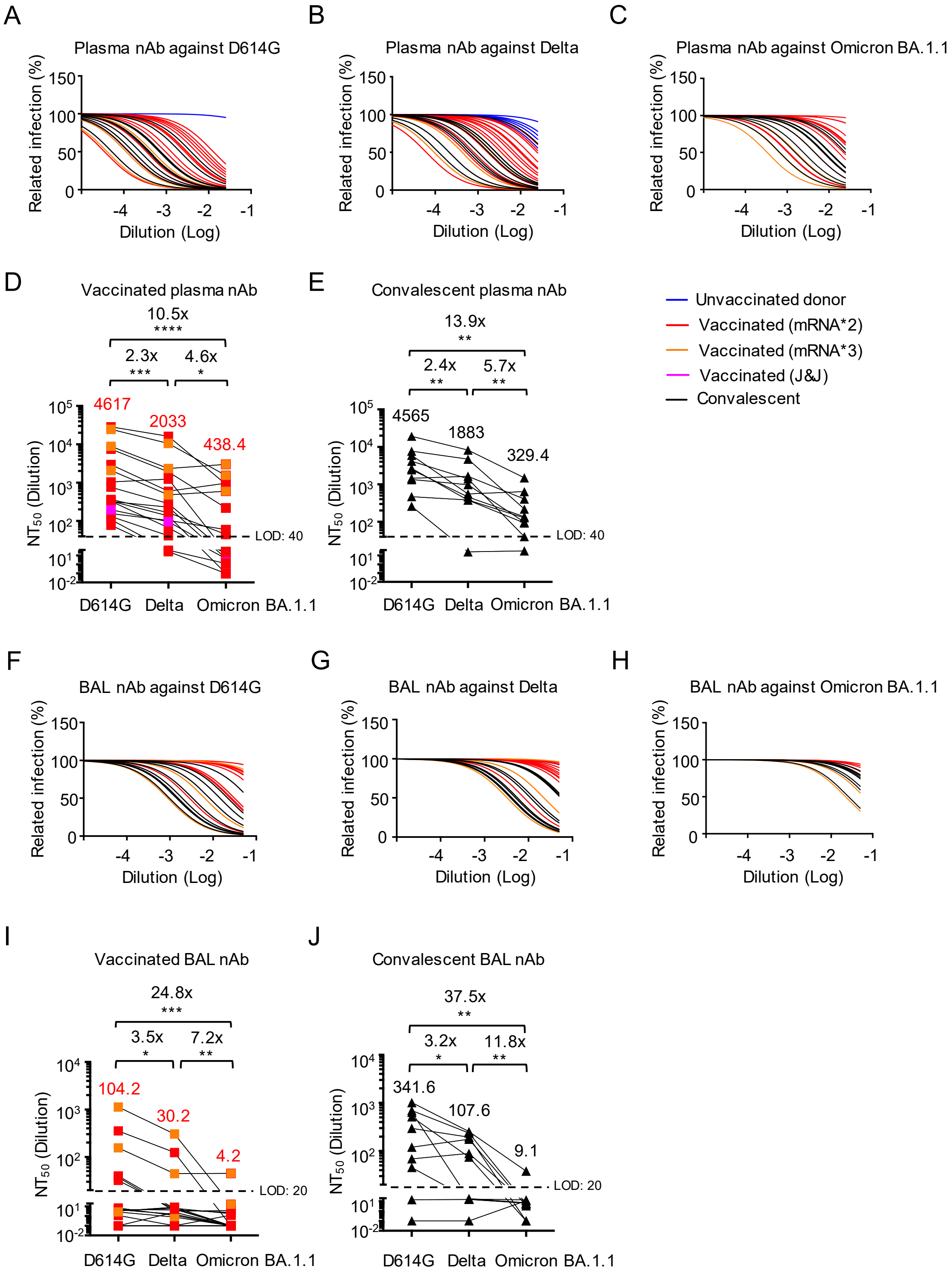

A
Plasma nAb against D614G
B
Plasma nAb against Delta
C
Plasma nAb against Omicron BA.1.1
Related infection (%)
Dilution (Log)
D
Vaccinated plasma nAb
10.5x
2.3x
4.6x
4617
2033
438.4
LOD: 40
NT50 (Dilution)
D614G Delta Omicron BA.1.1
E
Convalescent plasma nAb
13.9x
2.4x
5.7x
4565
1883
329.4
LOD: 40
Unvaccinated donor
Vaccinated (mRNA*2)
Vaccinated (mRNA*3)
Vaccinated (J&J)
Convalescent
F
BAL nAb against D614G
G
BAL nAb against Delta
H
BAL nAb against Omicron BA.1.1
I
Vaccinated BAL nAb
24.8x
3.5x
7.2x
104.2
30.2
4.2
LOD: 20
J
Convalescent BAL nAb
37.5x
3.2x
11.8x
341.6
107.6
9.1
LOD: 20

Fig. S2. SARS-CoV-2 neutralizing antibody responses in human plasma and BAL against D614G, Delta, and Omicron BA.1.1. (A to C) Related infection of SARS-CoV-2 S D614G (A), Delta (B) and Omicron BA.1.1 (C) pseudotyped virus with incubation of serially diluted plasma from unvaccinated donors, vaccinated and convalescent individuals. HEK293T-ACE2 cells used as targeted cells. **(D and E)** Neutralizing antibody titers (NT_{50}) of plasma against SARS-CoV-2 S D614G, Delta and Omicron BA.1.1 pseudotyped virus in vaccinated (D) and convalescent individuals (E). **(F to H)** Related infection of SARS-CoV-2 S D614G (F), Delta (G) and Omicron BA.1.1 (H) pseudotyped virus with incubation of serially diluted BAL from unvaccinated donors, vaccinated and convalescent individuals. HEK293T-ACE2 cells used as targeted cells. **(I and J)** Neutralizing antibody titers (NT_{50}) of BAL against SARS-CoV-2 S D614G, Delta and Omicron BA.1.1 pseudotyped virus in vaccinated (I) and convalescent individuals (J). Three individuals who received the booster (BNT162b2 or mRNA-1273) were indicated as orange in vaccinated group. n=5 for unvaccinated donors, n=17 for vaccinated, n=10 for convalescents. one received J&J vaccine was indicated as pink. Three individuals who received the booster (BNT162b2 or mRNA-1273) were indicated as orange. nAb, neutralizing antibody. LOD, limit of detection. Data in (D, E, I and J) are means ± SEM. Statistical differences were determined by one-way ANOVA and p values were indicated by ns, not significant ($P > 0.05$), * ($p < 0.05$), ** ($p < 0.01$), ***($p < 0.001$) and **** ($p < 0.0001$).

Figure S3

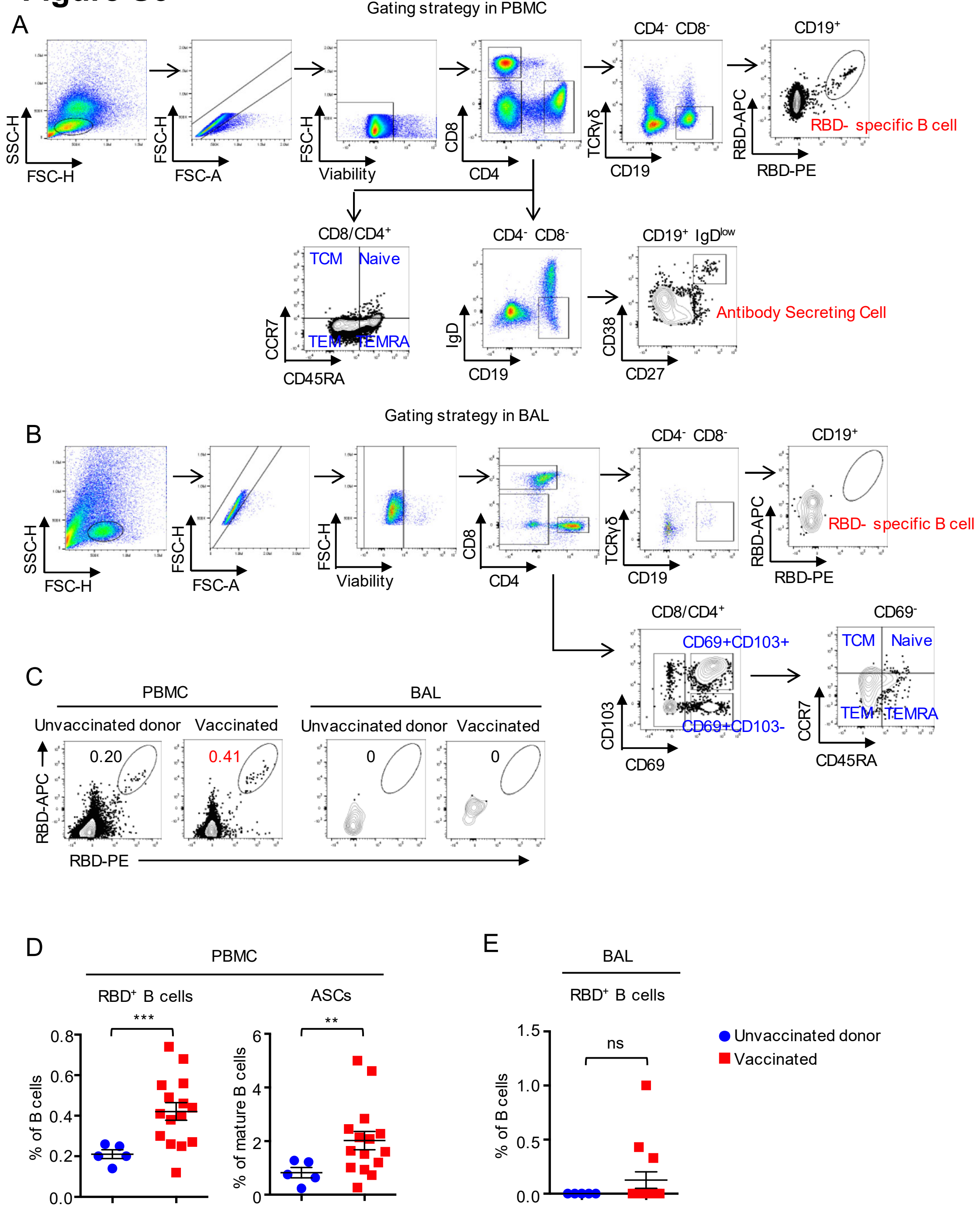

A
Gating strategy in PBMC
SSC-H
FSC-H
FSC-A
Viability
CD8
CD4
CD4- CD8-
TCRγδ
CD19
CD19+
RBD-APC
RBD-PE
RBD- specific B cell
CD8/CD4+
TCM
Naive
TEM
TEMRA
CCR7
CD45RA
IgD
CD19+ IgDlow
Antibody Secreting Cell
CD38
CD27
B
Gating strategy in BAL
CD69+CD103+
CD69+CD103-
CD103
CD69
CD69-
C
PBMC
BAL
Unvaccinated donor
Vaccinated
0.20
0.41
0
D
RBD+ B cells
ASCs
% of B cells
% of mature B cells
***
**
E
ns
Unvaccinated donor
Vaccinated

Fig. S3. **B cell response in human PBMC and BAL**. **(A and B)** Gating strategy of B cells and T cells in human PBMC (A) and BAL (B). **(C)** Representative flow cytometry plots of RBD- specific B cells in the PBMC and BAL from unvaccinated donors and vaccinated. **(D)** Frequencies of RBD- specific B cells and antibody- secreting cells (ASCs) in the PBMC from unvaccinated donors and vaccinated. **(E)** Frequencies of RBD- specific B cells in the BAL from unvaccinated donors and vaccinated. n=5 for unvaccinated donors, n=15 for vaccinated. TCM, central memory T cell. TEM, effector memory T cell, TEMRA, effector memory T cell re-expressing CD45RA. Data in D and E are means ± SEM. Statistical differences were determined by independent t test and p values were indicated by ns, not significant ($P > 0.05$), ** ($p < 0.01$), and *** ($p < 0.001$).

Figure S4

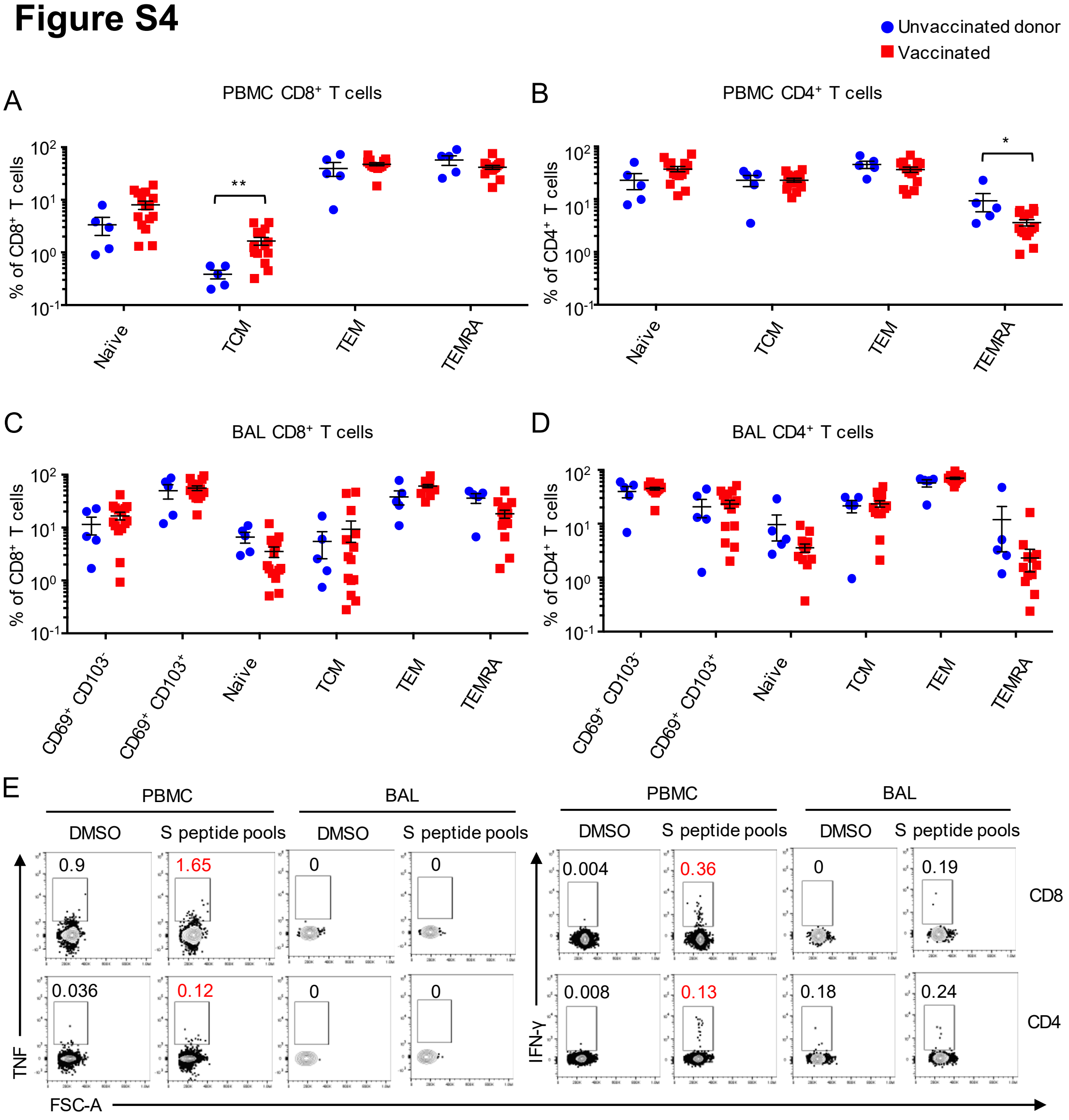

Fig. S4. **T cell response in human PBMC and BAL**. **(A and B)** Frequencies of CD8$^+$ (A) or CD4$^+$ (B) T cell subsets in the PBMC from unvaccinated donors and vaccinated. **(C and D)** Frequencies of CD8$^+$ (C) or CD4$^+$ (D) T cell subsets in the BAL from unvaccinated donors and vaccinated. **(E)** Representative flow cytometry plots of TNF- and IFN-γ- producing CD8$^+$ and CD4$^+$ T cells in the PBMC and BAL from unvaccinated donors and vaccinated after S peptide pools stimulation. n=5 for unvaccinated donors, n=15 for vaccinated. TCM, central memory T cell. TEM, effector memory T cell, TEMRA, effector memory T cell re-expressing CD45RA. Data in A to D are means ± SEM. Statistical differences were determined by independent t test and p values were indicated by * ($p < 0.05$) and ** ($p < 0.01$).

Figure S5

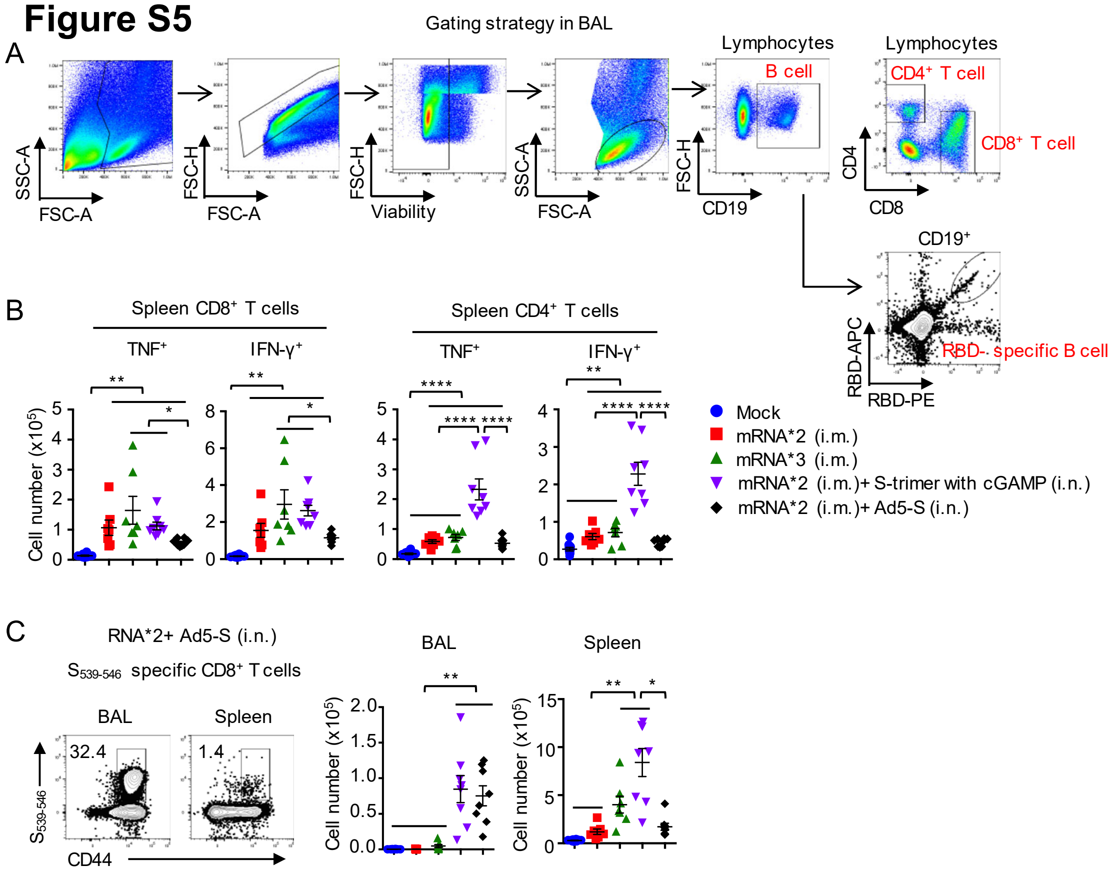

Fig. S5. T cell responses in mouse BAL and spleen. C57BL/6 mice were immunized as indicated immunization strategies. 14 days later, T cell responses were measured from BAL and spleen. **(A)** Gating strategy of B cells and T cells in the BAL. **(B)** Cell numbers of cytokines producing $CD8^+$ and $CD4^+$ T cells in the spleen after S peptide pools stimulation from different groups. **(C)** Representative flow cytometry plots of $CD44^+$ $S_{539-546}^+$ cells in $CD8^+$ T cells were presented as $S_{539-546}$ specific $CD8^+$ T cells (left panel); cell numbers of $S_{539-546}$ specific $CD8^+$ T cells in the BAL and spleen from two doses of RNA plus Ad5-S booster group (right panel). n=10 for Mock, n=7 for mRNA*2 (i.m.), n=7 for mRNA*3 (i.m.), n=8 for mRNA*2 (i.m.)+ S-trimer with cGAMP (i.n.), n=8 for mRNA*2 (i.m.)+ Ad5-S (i.n.). i.m., intramuscular. i.n., intranasal. Data are pooled from two independent experiments. Data in B and C are means ± SEM. Statistical differences were determined by one-way ANOVA and p values were indicated by * ($p < 0.05$), ** ($p < 0.01$) and **** ($p < 0.0001$).

Figure S6

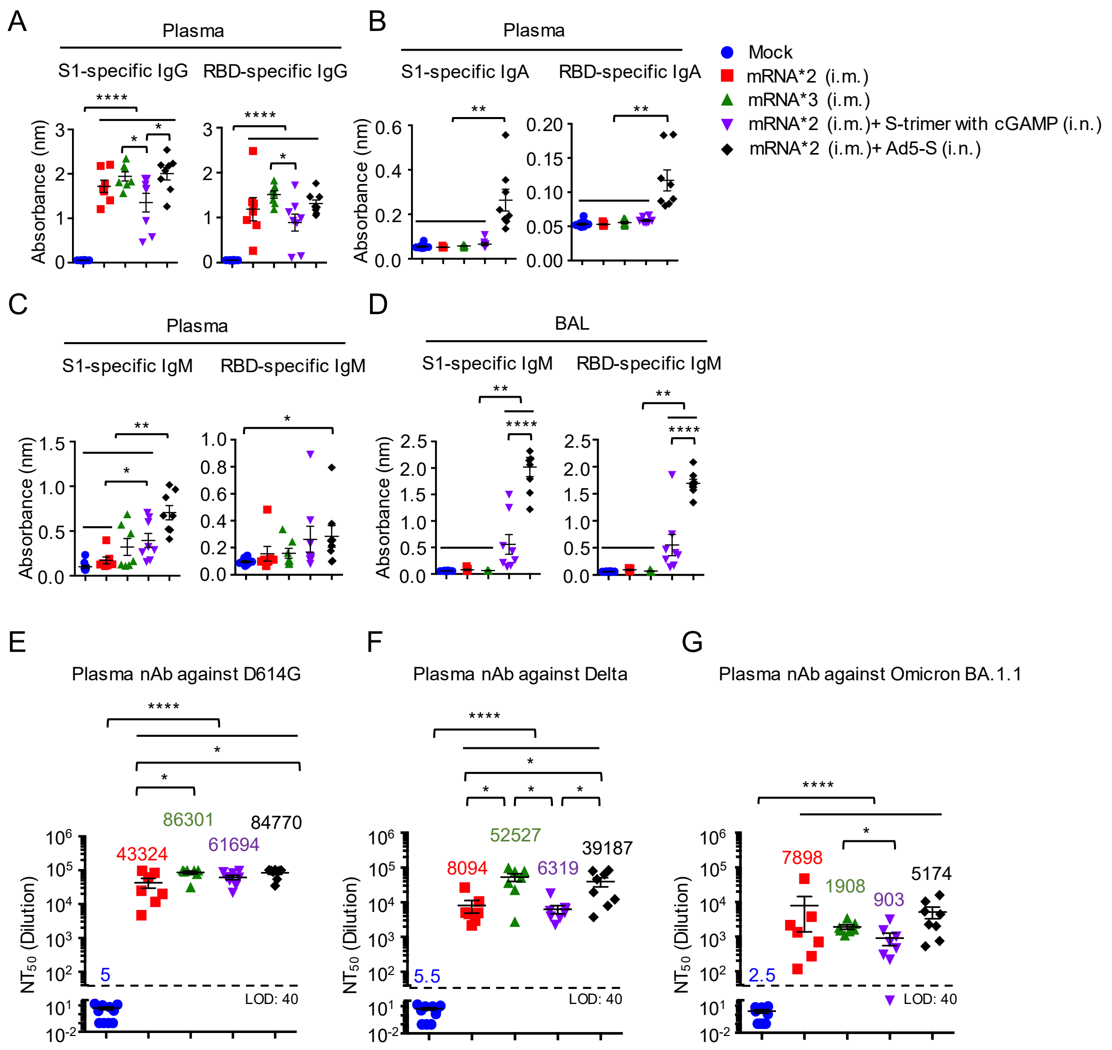

Fig. S6. Antibody responses in mouse plasma and BAL. C57BL/6 mice were immunized as indicated immunization strategies. 14 days later, binding antibody and neutralizing antibody responses were measured from plasma and BAL. **(A and B)** Levels of S1 or RBD- specific IgG (A) and IgA (B) were measured from plasma. **(C and D)** Levels of S1- specific IgM were measured from plasma (C) and BAL (D). **(E to G)** NT_{50} of plasma against SARS-CoV-2 S D614G (E), Delta (F) and Omicron BA.1.1 (G) pseudotyped virus were measured. nAb, neutralizing antibody. LOD, limit of detection. n=10 for Mock, n=7 for mRNA*2 (i.m.), n=7 for mRNA*3 (i.m.), n=8 for mRNA*2 (i.m.)+ S-trimer with cGAMP (i.n.), n=8 for mRNA*2 (i.m.)+ Ad5-S (i.n.). i.m., intramuscular. i.n., intranasal. nAb, neutralizing antibody. LOD, limit of detection. Data are pooled from two independent experiments. Data are means ± SEM. Statistical differences were determined by one-way ANOVA and p values were indicated by * ($p < 0.05$), ** ($p < 0.01$), and **** ($p < 0.0001$).

Figure S7

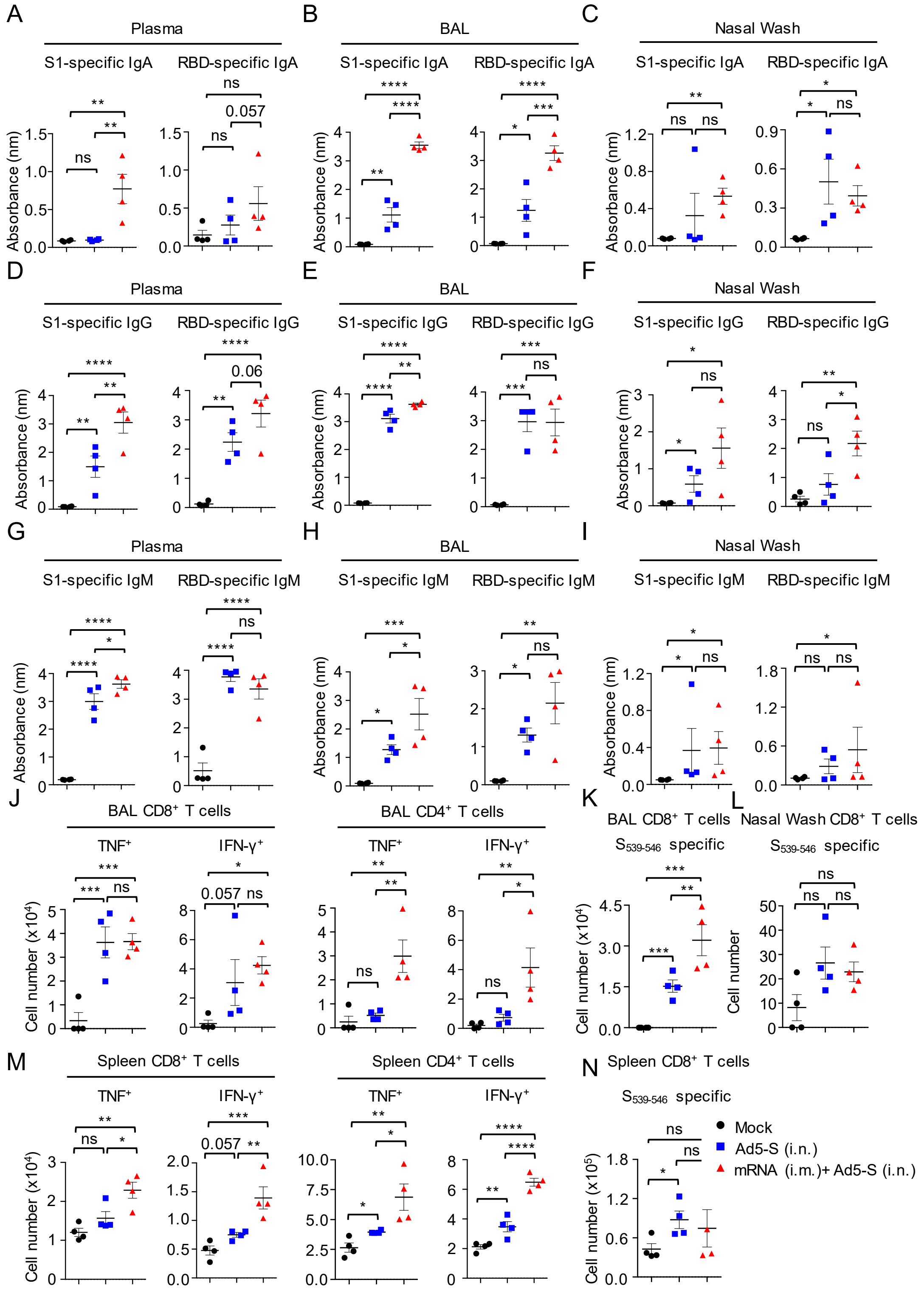
A
Plasma
S1-specific IgA
RBD-specific IgA
B
BAL
C
Nasal Wash
Absorbance (nm)
D
S1-specific IgG
RBD-specific IgG
E
F
G
S1-specific IgM
RBD-specific IgM
H
I
J
BAL CD8⁺ T cells
BAL CD4⁺ T cells
TNF⁺
IFN-γ⁺
Cell number (x10⁴)
K
BAL CD8⁺ T cells
S539-546 specific
L
Nasal Wash CD8⁺ T cells
S539-546 specific
Cell number
M
Spleen CD8⁺ T cells
Spleen CD4⁺ T cells
N
Spleen CD8⁺ T cells
S539-546 specific
Cell number (x10⁵)
Mock
Ad5-S (i.n.)
mRNA (i.m.)+ Ad5-S (i.n.)

Fig. S7. Antibody and T cell responses in mouse following mRNA-S plus Ad5-S or Ad5-S alone vaccination. C57BL/6 mice were immunized with PBS or mRNA-S at day 0, and then immunized with Ad5-S intranasally at day 21 as indicated. 14 days later, binding antibody response was measured from plasma, BAL and nasal wash. T cell response was measured from spleen, BAL and nasal wash. **(A to C)** Levels of S1- and RBD- specific IgA were measured from plasma (A), BAL (B) and Nasal wash (C). **(D to F)** Levels of S1- and RBD- specific IgG were measured from plasma (D), BAL (E) and Nasal wash (F). **(G to I)** Levels of S1- and RBD- specific IgM were measured from plasma (G), BAL (H) and Nasal wash (I). **(J to N)** Cell numbers of Spike-specific or cytokines producing $CD8^+$ and $CD4^+$ T cells in the BAL (J and K), Nasal wash (L) and spleen (M and N) from indicated groups. n=4 for each group. Data are means ± SEM. Statistical differences were determined by one-way ANOVA and p values were indicated by ns, not significant ($P > 0.05$), * ($p < 0.05$), ** ($p < 0.01$), *** ($p < 0.001$), and **** ($p < 0.0001$).

Figure S8

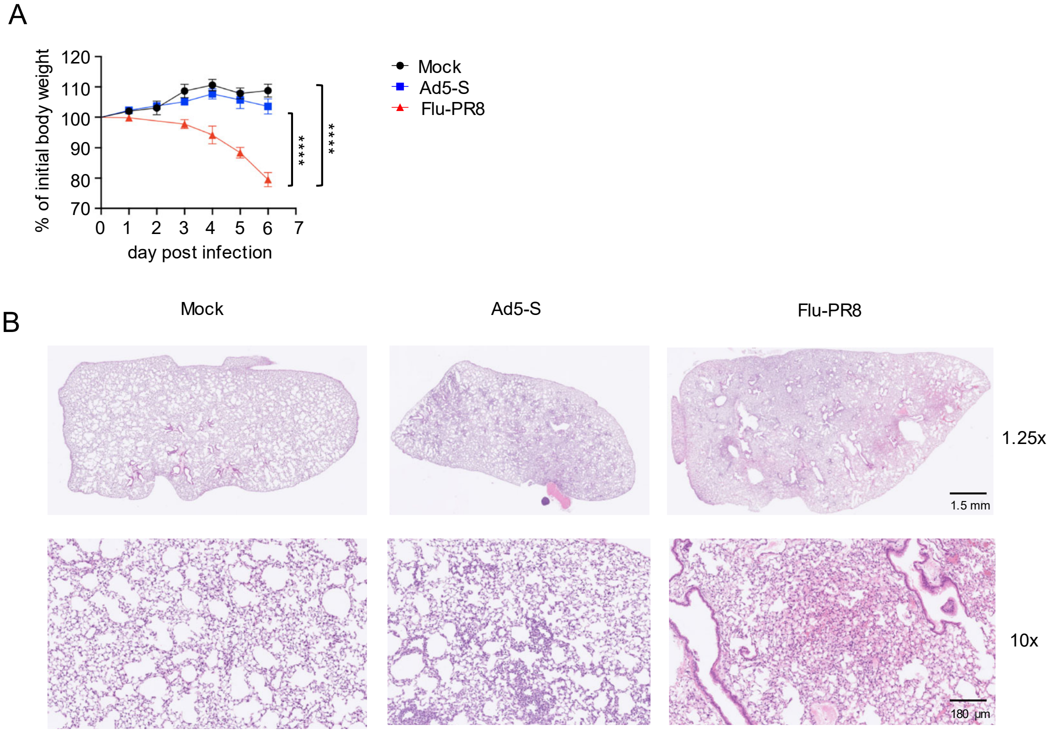

Fig. S8. Lung pathology following intranasal Ad5-S or influenza infection. (A) Percentage of initial mice body weight following Ad5-S (n=4), influenza (Flu-PR8) (n=5), or mock (n=4) infection. **(B)** Lung histology by H&E staining at 6 days post infection. Data in (A) are means ± SEM. Statistical differences were determined by one-way ANOVA and p values were indicated by **** ($p < 0.0001$).

Figure S9

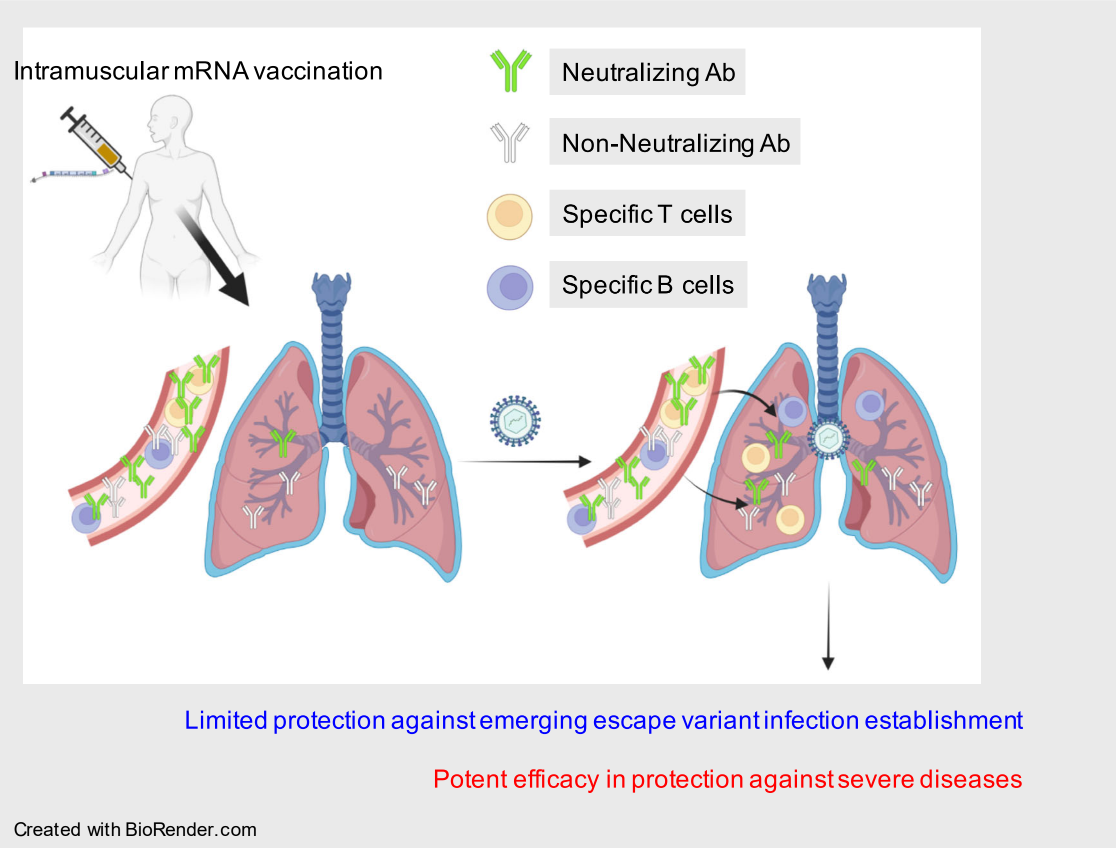

Fig. S9. Model on the mechanism of protection by intramuscular mRNA vaccine-induced adaptive immunity against severe diseases, rather than infection. In this model, robust neutralizing Ab, specific T cell and B cell responses were detected in circulating blood but limited in the respiratory tract post intramuscular mRNA vaccination. Adaptive immunity in local site failed to prevent respiratory infection by escape variants, but potently protect against severe diseases with the recruitment of systemic humoral and cellular immunity into the infection site upon SARS-CoV-2 escape variant infection.

Table S1. Enrolled donors

ID number	Sex	Age	Vaccine received	Third dose	Day post last vaccination	Nucleocapsid antibodies
Vac1	M	63	PFIZER/ BIONTECH	N	51	N
Vac2	M	67	MODERNA	N	71	N
Vac3	M	78	MODERNA	N	92	N
UD1	F	69	NONE	N/A	N/A	N
UD2	F	74	NONE	N/A	N/A	N
Vac4	F	77	MODERNA	N	87	N
Vac5	F	64	PFIZER/ BIONTECH	N	124	N
Vac6	M	73	JANSSEN (J&J)	N	107	N
Vac7	M	39	PFIZER/ BIONTECH	N	95	N
Vac8	M	62	PFIZER/ BIONTECH	N	104	N
Vac9	F	85	MODERNA	N	187	N
Vac10	F	75	MODERNA	N	206	N
Vac11	F	73	MODERNA	N	165	N
Vac12	M	62	PFIZER/ BIONTECH	N	171	N
Vac13	F	74	MODERNA	N	235	N
Vac14	F	48	PFIZER/ BIONTECH	N	206	N
Vac15	M	78	MODERNA	Y	34	N
Vac16	M	61	PFIZER/ BIONTECH	Y	57	N
Vac17	M	73	mRNA (not sure the brand)	N	73	N
Vac18	M	69	PFIZER/ BIONTECH	Not sure	[illegible] 165	N
Vac19	M	62	MODERNA	Y	51	N

Vac4 and Vac5 lack blood samples; Vac6 and Vac7 lack BAL samples

Continued

ID number	Sex	Age	COVID-19 confirmation	Vaccine received	Day post diagnosis	Symptoms at enrollment	Nucleocapsid antibodies
UD3	M	60	N	NONE	N/A	N/A	N
UD4	F	77	N	NONE	N/A	N/A	N
UD5	M	73	N	NONE	N/A	N/A	N
Conv1	M	83	Y	NONE	74	Cough	P
Conv2	M	63	Y	NONE	74	Fatigue	P
Conv3	M	65	Y	NONE	88	mild dyspnea/Fatigue	P
Conv4	M	74	Y	NONE	74	Moderate dyspnea	P
Conv5	F	66	Y	NONE	87	Mild dyspnea	P
Conv6	M	62	Y	NONE	84	N	P
Conv7	F	76	Y	mRNA (not sure the brand)	80	Severe dyspnea/ Fatigue	P
Conv8	F	63	Y	NONE	78	Severe dyspnea/ Fatigue/Cough	P
Conv9	M	68	Y	NONE	67	N	P
Conv10	F	64	Y	Not sure	66	Not sure	P

Table S2. List of human antibodies for flow cytometry

ANTIGEN	FLUORESCENCE	CLONE	CATALOGUE NUMBER	COMPANY
CD103	APC/Fire750	Ber-ACT8	350238	Biolegend
CD19	BV786	SJ25C1	664532	BD Biosciences
CD27	Pacific Blue	M-T271	356414	Biolegend
CD38	BV480	HIT2	566186	BD Biosciences
CD4	AF532	SK3	58-0047-42	ThermoFisher Scientifics
CD4	BV421	RPA-T4	300532	Biolegend
CD45RA	BV650	HI100	304136	Biolegend
CD69	APC-Cy7	FN50	310914	Biolegend
CD69	PE-eFluor610	FN50	61-0699-42	ThermoFisher Scientifics
CD8	PerCP-eFluor710	OKT8	46-0086-42	ThermoFisher Scientifics
CD8	FITC	OKT8	11-0086-42	ThermoFisher Scientifics
CCR7	BV711	G043H7	353228	Biolegend
IFN-γ	PE	B27	562016	BD Biosciences
IgD	BV510	IA6-2	348220	Biolegend
TCRγδ	BV750	11F2	747127	BD Biosciences
TNF-α	AF700	MAb11	56-7349-42	ThermoFisher Scientifics
Viability	Zombie Aqua		423102	Biolegend
Viability	Zombie NIR		423106	Biolegend

Table S3. List of mouse antibodies for flow cytometry

ANTIGEN	FLUORESCENCE	CLONE	CATALOGUE NUMBER	COMPANY
CD103	BV605	2E7	121433	Biolegend
CD38	BV421	90	102732	Biolegend
CD4	AF700	GK1.5	100430	Biolegend
CD44	BV421	IM7	103040	Biolegend
CD44	BV510	IM7	103044	Biolegend
CD69	FITC	H1.2F3	104506	Biolegend
CD8a	BV711	53-6.7	100759	Biolegend
B220	BV711	RA3-6B2	103255	Biolegend
IFN-γ	BV605	XMG1.2	505840	Biolegend
GL-7	AF488	GL7	144612	Biolegend
TNF-α	PE-Cy7	MP6-XT22	506324	Biolegend
Viability	Zombie Aqua		423102	Biolegend
Viability	Zombie NIR		423106	Biolegend
